# Model based development of tacrolimus dosing algorithm considering CYP3A5 genotypes and mycophenolate mofetil drug interaction in stable kidney transplant recipients

**DOI:** 10.1038/s41598-019-47876-0

**Published:** 2019-08-13

**Authors:** Jae Hyun Kim, Nayoung Han, Myeong Gyu Kim, Young Won Kim, Hayoung Jang, Hwi-Yeol Yun, Mi-Yeon Yu, In-Wha Kim, Yon Su Kim, Jung Mi Oh

**Affiliations:** 1 College of Pharmacy and Research Institute of Pharmaceutical Sciences, Seoul National University, Seoul, Republic of Korea; 2 Graduate School of Clinical Pharmacy, CHA University, Pocheon, Republic of Korea; 3 College of Pharmacy, Chungnam National University, Daejeon, Republic of Korea; 4 Department of Internal Medicine, Hanyang University Guri Hospital, Guri, Republic of Korea; 5 Kidney Research Institute, Seoul National University College of Medicine, Seoul, Republic of Korea; 6 Department of Medical Science, Seoul National University College of Medicine, Seoul, Republic of Korea

**Keywords:** Immunosuppression, Combination drug therapy

## Abstract

This study quantifies the interaction between tacrolimus (TAC) and mycophenolate mofetil (MMF) in kidney transplant recipients. Concentrations of TAC, mycophenolic acid (MPA), and metabolites were analyzed and relevant genotypes were determined from 32 patients. A population model was developed to estimate the effect of interaction. Concentrations of TAC were simulated in clinical scenarios and dose-adjusted trough concentrations per dose (C/D) were compared. Effect of interaction was described as the inverse exponential relationship. Major determinants of trough levels of TAC were *CYP3A5* genotype and interaction with MPA. The absolute difference in C/D of TAC according to co-administered MMF was higher in *CYP3A5* non-expressers (0.55 ng/mL) than in *CYP3A5* expressers (0.35 ng/mL). The effect of MMF in determining the TAC exposure is more pronounced in *CYP3A5* non-expressers. Based on population pharmacokinetic model, we suggest the TAC dosing algorithm considering the effects of *CYP3A5* and MMF drug interaction in stable kidney transplant recipients.

## Introduction

Tacrolimus (TAC), the backbone of immunosuppressive drug therapy in organ transplantation, is mostly used in combination with mycophenolate mofetil (MMF) as a maintenance immunosuppressive regimen to prevent graft rejection and improve overall graft and patient survival in kidney transplant recipients^1,2^. However, TAC is characterized by a narrow therapeutic index and a considerably high intra- and inter-individual variability (IIV) in pharmacokinetics, resulting in fairly different drug concentration-time profiles over time^3^. This hinders the precise prediction of dosage requirement necessary for optimal clinical outcomes.

Several factors have been identified to influence the pharmacokinetic variability of TAC which include body weight, hematocrit (HCT), aspartate aminotransferase (AST), alanine aminotransferase (ALT), gastrointestinal function, post-operative days (POD), steroid use, and drug-drug interaction (DDI)^4–8^. Genotypes including *CYP3A4*, *CYP3A5*, and *ABCB1* were also identified as important factors for pharmacokinetics of TAC^9–11^.

Although the glucuronidation is more involved in the major metabolic pathway of mycophenolic acid (MPA), TAC and MPA are both substrates for CYP3A enzymes and P-glycoprotein (Pgp)^12,13^. Co-administered drugs that inhibit or induce these mechanisms may increase or decrease the blood concentrations of TAC^13–15^. Researches have also independently observed the association between the genetic polymorphisms of enzymes or transporters and the concentrations of TAC and MMF^16–19^. As the genetic variations of the respective enzymes and transporters are known to affect the trough levels and ultimately the dosage requirements in transplant patients, it is important to know the effect of genetic polymorphism on the pharmacokinetic variability^20,21^.

These all suggest that a detailed knowledge of the potential pharmacokinetic interaction between TAC and MMF and its magnitude are of great importance in understanding of the variability of TAC pharmacokinetics, which is crucial in the clinical management of kidney transplant patients. However, the information on the sources of variability is limited, especially on the effects of co-administered MMF on the pharmacokinetics of TAC.

Interaction potential between TAC and MMF has been raised based on the results of scarce *in vitro* or clinical studies^14,22,23^. Our previous study of a population pharmacokinetic drug interaction model demonstrated that the area under the concentration-time curve (AUC) of TAC increased by 22.1% when co-administered with MMF in healthy volunteers^23^. Effect of interaction also differed according to *CYP3A5* genotypes^23^. This suggests that lower doses of TAC are required to achieve adequate immunosuppression when used with MMF. However, as the result of the study was obtained from healthy volunteers after a single dose of TAC and MMF, the effect of interaction cannot be generalized to stable kidney transplant recipients. More importantly, pharmacokinetic data from TAC administered as a single agent cannot be used to support the dose rationale in its combination therapy with MMF.

As the concentration of TAC is closely related to graft survival^24,25^, it is important to understand the relevant factors including concomitant drug administration, which influence the variability of TAC and to quantify their effects on the concentration of TAC to assist in drug dosage decisions in patients.

Population pharmacokinetic models have been widely employed to explore the factors of variability, which are incorporated into models as covariate factors. A population pharmacokinetic model that can quantify the impact of the DDI and identify the covariates that contribute to the drug levels of TAC is needed to establish the rationale for its dose adjustments in kidney transplant patients receiving MMF concomitantly as a combination maintenance regimen. The integrated pharmacokinetic model of TAC and MMF enables the exploration of dynamic changes in the concentrations of both drugs with accounting the effects of interaction or other covariates at the same time.

Therefore, this study aimed to explore a population pharmacokinetic model of TAC to identify and quantify the clinical and genetic covariates that explain the variability to ensure appropriate dosing recommendations for those who require TAC as a combination regimen with MMF in stable kidney transplant recipients.

## Results

### Demographics

A total of 32 kidney transplant recipients were enrolled in the study (Table 1). The median age was 52 (20–70) years and 20 patients (63%) were male. All patients were Korean. Median post-operative days was 5.7 years (range 0.6–10.4 years). Levels of HCT (range 32.3–53.8%) and estimated glomerular filtration rate (eGFR) calculated by Modification of Diet in Renal Disease (MDRD) (range 35.0–85.7 mL/min/1.73 m^2^) were in the near-normal ranges. The median daily dose of TAC and MMF were 2 mg and 1,000 mg, respectively. Thirteen patients (40.6%) were *CYP3A5* expressers (CYP3A5*1/*1 or CYP3A5*1/*3 carriers). For *SLCO1B3* rs4149117 and *UGT2B7* rs7439366 genotypes, fifteen patients (46.9%) were T carriers.

**Table 1:** Baseline characteristics of included patients (*n* = 32).

Characteristics	Median	Range (min–max)
Age (yr)	52	20–70
Sex (male)^a^	20	63%
Weight (kg)	62.7	43.9–102.4
Height (cm)	165.8	151–180
Hemoglobin (g/dL)	14.1	10.6–17.5
Hematocrit (%)	43.6	32.3–53.8
Serum creatinine (mg/dL)	1.22	0.69–1.66
MDRD eGFR (mL/min/1.73 m^2^)	59.5	35.0–85.7
Albumin (g/dL)	4.4	3.8–5.0
Total bilirubin (mg/dL)	0.7	0.5–2.4
Tacrolimus daily dose (mg)	2	1–6
Mycophenolate mofetil daily dose (mg)	1,000	500–2,000
Prednisolone daily dose (mg)^b^	5	2.5–5
Post-operative days (yr)	5.7	0.6–10.4
Collected blood samples per patients	4	1–5
*CYP3A5* (rs776746) expresser^a^	13	40.6%
*SLCO1B3* (rs4149117) T carrier^a^	15	46.9%
*UGT2B7* (rs7439366) T carrier^a^	15	46.9%

^a^Sex and *CYP3A5* expresser are presented as number and proportion; ^b^Prednisolone or its equivalent; *CYP3A5* expresser, *CYP3A5* *1/*1 or *CYP3A5* *1/*3 carriers; MDRD, modification of diet in renal disease; eGFR, estimated glomerular filtration rate.

### Population pharmacokinetic model development

Pharmacokinetic parameter estimates, relative standard error (RSE), IIV in parameter estimates of a developed model are presented in Table 2. CL*/F* and volume of distribution of TAC were estimated as 21.9 L/h (RSE 16%) and 103 L (RSE 15%), respectively. A combined additive and proportional model was used to account for the residual error of the developed model. The only significant covariate identified for the pharmacokinetic parameters of TAC was *CYP3A5* genotype. In *CYP3A5* expressers, CL*/F* of TAC was increased to 1.49-fold (RSE 9%) compared to *CYP3A5* non-expressers (Eq. 1).

**Table 2:** Population pharmacokinetic parameter estimates of models for TAC and MMF.

Parameter	Base model		Final model	
	Population mean value (%RSE)	IIV CV% (%RSE)	Population mean value (%RSE)	IIV CV% (%RSE)
***Tacrolimus***				
*CL*_TAC_ (L/h)	28.5 (19%)	31.3% (13%)	21.9 (16%)	24.4% (14%)
*V*_TAC_ (L)	102 (15%)	46.8% (16%)	103 (15%)	45.5% (18%)
*K*_*a* TAC_ (h^−1^)	1.78 FIX	—	1.78 FIX	—
*k*_23_ (h^−1^)	0.863 (47%)	—	0.803 (44%)	—
*k*_32_ (h^−1^)	0.641 (48%)	—	0.583 (51%)	—
Lag time (h)	0.59 FIX	—	0.59 FIX	—
*CYP3A5 on CL*_TAC_	—	—	1.49 (9%)	—
*σ*_add TAC_	0.687 (34%)	—	0.585 (48%)	—
*σ*_prop TAC_	0.203 (13%)	—	0.211 (14%)	—
***Mycophenolic acid***				
*CL*_MPA_ (L/h)	2.94 (9%)	24.8% (15%)	3.27 (10%)	25.8% (17%)
*V*_MPA_ (L)	22.9 (33%)	42.9% (18%)	23.2 (33%)	42.9% (19%)
*K*_*a* MPA_ (h^−1^)	2.29 FIX	—	2.29 FIX	—
*k*_56_ (h^−1^)	1.71 (24%)	—	1.75 (23%)	—
*k*_65_ (h^−1^)	0.0081 (17%)	—	0.0089 (18%)	—
*k*_70_ (h^−1^)	0.0958 (21%)	—	0.103 (18%)	—
*V*_MPAG_ (L)	1.82 (17%)	—	1.76 (15%)	—
f _MPA_	0.85 FIX	—	0.85 FIX	—
EHC (%)	0.367 FIX	—	0.367 FIX	—
*k*_84_ (h^−1^)	18.4 FIX	—	18.4 FIX	—
MTIME1	7.96 FIX	—	7.96 FIX	—
MTIME2	1 FIX	—	1 FIX	—
*V*_AcMPAG_ (L)	13.9 FIX	—	13.9 FIX	—
*k*_90_ (h^−1^)	0.407 FIX	—	0.407 FIX	—
*UGT2B7 on k*_59_	—	—	0.812 (10%)	—
*SLCO1B3 on V*_MPAG_ (L)	—	—	1.2 (10%)	—
*σ*_prop MPA_	0.507 (7%)	—	0.515 (7%)	—
*σ*_prop MPAG_	0.221 (8%)	—	0.216 (8%)	—
*σ*_add AcMPAG_	0.0719 (25%)	—	0.0647 (23%)	—
*σ*_prop AcMPAG_	0.284 (9%)	—	0.268 (8%)	—
Interaction	0.0732 (39%)	—	0.06 (35%)	—

IIV, interindividual variability; CV, coefficient of variation; RSE, relative standard error; F, fraction of the dose absorbed; CL, clearance; TAC, tacrolimus; V, volume of distribution; *K*_a_, first-order absorption rate constant; *k*_23_, *k*_32_, *k*_56_, and *k*_65_, intercompartment rate constants; MPA, mycophenolic acid; *k*_70_ and *k*_90_, eliminated rate constants; *CYP3A5*, *CYP3A5* expressers (CYP3A5*1/*1 or CYP3A5*1/*3 carriers); f_MPA_, fraction of MPA which metabolized to MPAG; EHC, enterohepatic circulation; *k*_84_, gallbladder emptying rate constant; MTIME1, meal time; MTIME2, Gallbladder emptying duration; MPAG, MPA 7-O-glucuronide; AcMPAG, MPA acyl glucuronide; *SLCO1B3*, *SLCO1B3* rs4149117 T carrier; *UGT2B7*, *UGT2B7* rs7439366 T carrier; σ_prop_, proportional residual error; σ_add_, additive residual error.

The elimination rate constant (*k*_90_) and volume of distribution of MPA acyl glucuronide (AcMPAG) were once estimated in kidney transplant recipients and then fixed to estimated values thereafter. Genotypes of *SLCO1B3* and *UGT2B7* were identified as significant covariates that influence the pharmacokinetics of MMF. In T carriers of *SLCO1B3* 334T > G (rs4149117), the volume of distribution of MPA 7-O-glucuronide (MPAG) was increased to 1.2-fold (RSE 10%) compared to GG genotype. In T carriers of *UGT2B7* 802T > C (rs7439366), formation rate of AcMPAG was decreased to 0.8-fold (RSE 10%) compared to CC genotype.

The interaction effect was modeled as an inverse exponential relationship between the CL*/F* of TAC and the concentration of MPA (C_MPA_) (Eq. 1). The slope parameter which mediates the effect of interaction was estimated as 0.06 (RSE 35%). Inclusion of DDI effect on the CL*/F* of TAC led to stabilization of model parameter estimates. When the effect of DDI in the full model is removed through the backward elimination procedure, the objective function value (OFV) increases from 1090.6 to 1102.1 (ΔOFV = 11.5). IIV in CL*/F* of TAC also increases by 9% from 24.3% to 33.3% without the effect of DDI.

1 $${\rm{C}}{\rm{L}}/F\,of\,TAC=21.9\times \frac{1}{{e}^{0.06\cdot {C}_{MPA}}}\times {1.49}^{CYP3A5}$$

CL*/F* (L/h); C_MPA_ (µg/mL); the value of *CYP3A5* is 1 in *CYP3A5* expressers, and 0 in otherwise

### Model Evaluation

**Figure 1:**
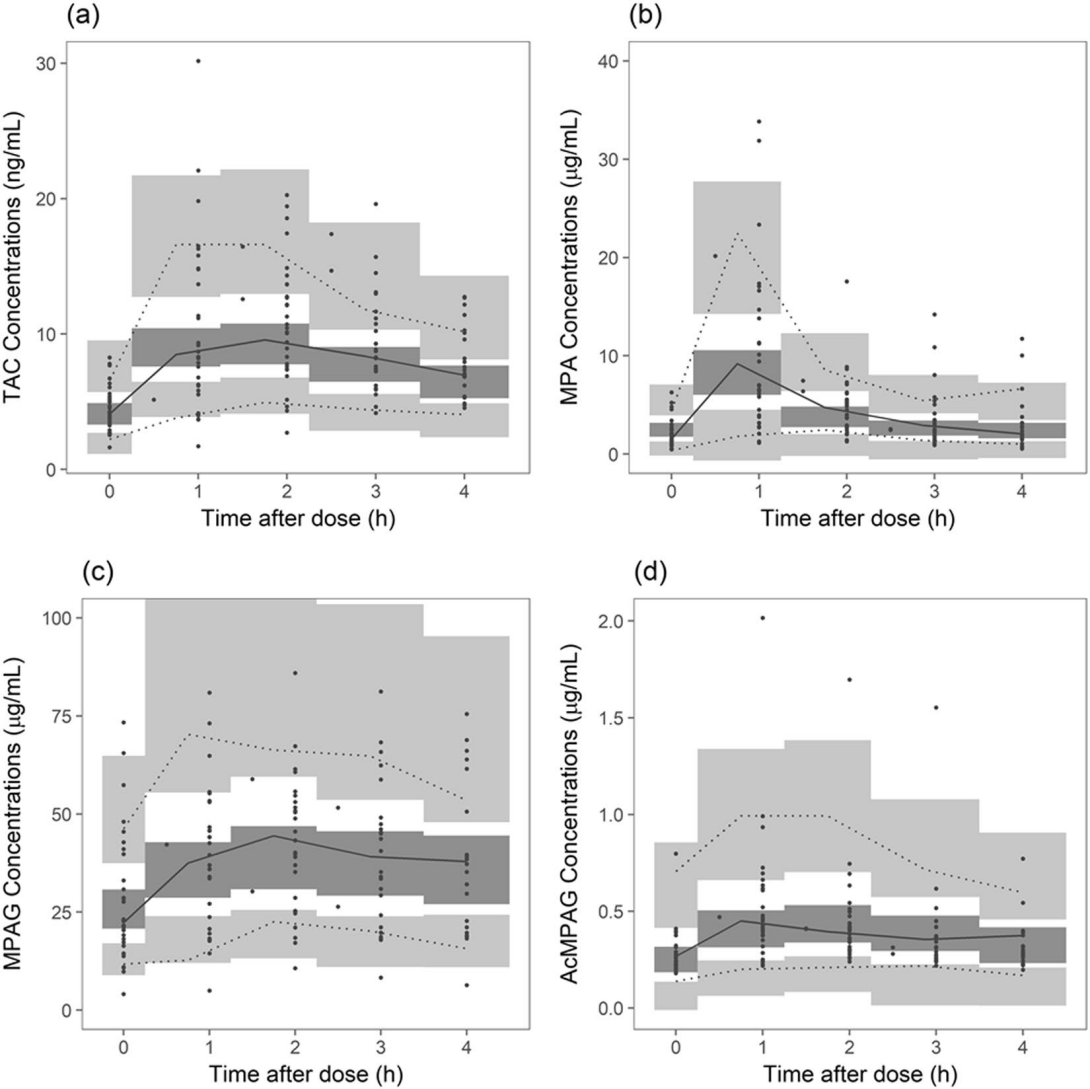
Prediction-corrected visual predictive check of population pharmacokinetic models. (**a**) TAC; (**b**) MPA; (**c**) MPAG; (**d**) AcMPAG. Closed circles represent observed concentrations. Solid red line represents median of observed concentrations and solid blue line represents 5^th^ and 95^th^ percentile of observed concentrations. Blue area represents 95% confidence interval of 5^th^ and 95^th^ percentile of predicted concentrations and red area, 95% confidence interval of the median of predicted concentrations. TAC, tacrolimus; MPA, mycophenolic acid; MPAG, MPA 7-O-glucuronide; AcMPAG, MPA acyl glucuronide

Goodness-of-fit of TAC of the final population pharmacokinetic model is presented in Figs S1–S4. After IIV in pharmacokinetics was accounted for, improvement in model fitting was noted. Distribution of conditional weighted residuals (CWRES) was not different according to time or population prediction and showed no evidence of model misspecification. Prediction-corrected visual predictive check (VPC) plots of TAC, MPA, MPAG, and AcMPAG are presented in Fig. 1. Observed concentrations were overlaid in the confidence interval (CI) of model predicted concentrations, which assures the predictive performance of the final model.

### Simulation

Steady state concentrations of TAC were simulated in 32 virtual populations. Each population was generated with the combination of the dose of TAC (1 mg bid and 2 mg bid), the dose of MMF (500 mg bid and 1,000 mg bid), *CYP3A5* genotype (expresser and non-expresser), *SLCO1B1* genotype (T carrier and GG genotype), and *UGT2B7* genotype (T carrier and CC genotype). Trough levels of TAC were higher in the combination of *CYP3A5* non-expressers and the higher dose of MMF. Trough concentrations per total daily dose (C/D) of TAC was increased from 0.89 ± 0.44 ng/mL to 1.24 ± 0.59 ng/mL in *CYP3A5* expressers and from 1.59 ± 0.67 ng/mL to 2.14 ± 0.88 ng/mL in *CYP3A5* non-expressers, respectively. Effect of *SLCO1B3* and *UGT2B7* genotypes on C/D of TAC was insignificant.

According to the final model and the simulation results, detailed dosing algorithm of TAC was developed. Under the possible combinations of *CYP3A5* genotype and the dose of MMF, required dose of TAC is presented in Table 3. Daily dose of 1–6 mg of TAC was required to achieve the target trough concentration, which is in consistent of administered dose range of TAC in patient population. When the patient’s genotype is *CYP3A5* expresser or the dose of MMF is 250 mg twice daily, larger amount of TAC was needed to achieve target level.

**Table 3:** Suggested algorithm on tacrolimus dosing based on *CYP3A5* and the dose of mycophenolate mofetil.

Target trough level of TAC	*CYP3A5* expressers				*CYP3A5* non-expressers			
	MMF 250 mg bid	MMF 500 mg bid	MMF 750 mg bid	MMF 1 g bid	MMF 250 mg bid	MMF 500 mg bid	MMF 750 mg bid	MMF 1 g bid
3–4 ng/mL	2 mg bid	1.5 mg bid	1 mg bid	1 mg bid	1 mg bid	1 mg bid	0.5 mg bid	0.5 mg bid
4–5 ng/mL	2.5 mg bid	2 mg bid	2 mg bid	1 mg bid	1.5 mg bid	1 mg bid	1 mg bid	0.5 mg bid
5–7 ng/mL	3 mg bid	2.5 mg bid	2 mg bid	1.5 mg bid	2 mg bid	1.5 mg bid	1 mg bid	1 mg bid

bid, twice daily; *CYP3A5* expresser, CYP3A5*1/*1 or CYP3A5*1/*3 carriers; *CYP3A5* non-expresser, CYP3A5*3/*3 carriers; MMF, mycophenolate mofetil; TAC, tacrolimus.

## Discussion

The present study was the first study to evaluate the effect of DDI between TAC and MMF in stable kidney transplant recipients with the integrated population pharmacokinetic model and further suggest the dosing algorithm of TAC. Although TAC is frequently co-administered with MMF, the magnitude of the interaction effect was unclear and the dosing recommendation with regards to the effect of interaction was scarce. The effect of co-administered MMF and other clinical factors including the dose of TAC and genotypes (*CYP3A5*, *SLCO1B3*, *and UGT2B7*) on the concentration of TAC was evaluated with modelling and simulation. The absolute difference in C/D of TAC according to dose increment of co-administered MMF was higher in *CYP3A5* non-expressers than in *CYP3A5* expressers (0.35 ng/mL in *CYP3A5* expressers vs. 0.55 ng/mL in *CYP3A5* non-expressers).

In this research, population pharmacokinetic model-based evaluation was used to simultaneously quantify the effect of DDI, genetic polymorphism, and other known clinical factors on the pharmacokinetics of TAC. The pharmacokinetic model development approach has additional strengths in that researchers can simulate various clinical scenarios based on combinations of identified clinical covariates. A number of studies have proven pharmacokinetic model development as an appropriate approach to elucidate the effect of DDI and/or genotypes^26–28^.

In the final population pharmacokinetic model, *CYP3A5* genotype was a significant covariate with respect to the CL*/F* of TAC. Historically, *CYP3A5* genotypes were repetitively identified as significant factors in previous population pharmacokinetic studies^5^. As *CYP3A5* is involved in the major metabolic pathway of TAC, and MPA showed possible competition for *CYP3A* in the previous *in vitro* study^14^, there is a potential for pharmacokinetic interaction. In our research, simulation of the final model revealed that effect of *CYP3A5* genotype was more influential in determining the trough levels of TAC than co-administration with MMF. In a previous study which evaluated the interaction between TAC and azole antifungals, the interaction effect differed according to *CYP3A5* genotype and was also blunted in *CYP3A5* expressers^29^. Because the trough concentration of TAC itself is lower in *CYP3A5* expressers, the effect of interaction is observed to be greater in *CYP3A5* non-expressers. Therefore, more caution is required when the dose of TAC is adjusted in *CYP3A5* non-expressers.

For the population pharmacokinetic model of mycophenolic acid and its metabolites, the population estimate of CL*/F* of MPA was 3.27 L/h. Low estimate of CL*/F* of MPA, ranging between 10.2–18.3 L/h has been observed in population pharmacokinetic models with Asian population^30,31^. Researchers suggest the need for lower dose of MMF in Asian population based on observed lower CL*/F*^32^. Another population pharmacokinetic model showed reduced CL*/F* of MPA (2.87 L/h) in renal transplant recipients with corticosteroid-free regimen^33^. Although all patients in our model were on steroids, fourteen of them received less than 5 mg/day of prednisolone or its equivalent. Ethnic difference, the dose of co-administered corticosteroids, and limited sampling points might be collectively related to relatively low CL*/F* compared to other previous studies^30,31,33^.

Genotypes *SLCO1B3* and *UGT2B7* were included as significant covariates for the volume of distribution of MPAG and formation rate of AcMPAG, respectively. The effect of identified genotype covariates was consistent with previous functional studies. In a study by Picard, *et al*., hepatic uptake of MPAG was increased and the dose-normalized concentration of MPAG was decreased in *SLCO1B3* 334T > G (rs4149117) T carriers^19^. Regarding *UGT2B7*, the AcMPAG metabolic rate was lower in T carriers of *UGT2B7* 802C > T (rs7439366) genotype in an *in vitro* human liver microsome study than in C homozygote genotype^34^. Although the pharmacokinetic model of MPA was statistically improved by considering the effect of *SLCO1B3* and *UGT2B7* genotypes, simulated trough concentrations of TAC were not affected.

In the final model, the concentrations of MPA were linked to the CL*/F* of TAC with an inverse exponential equation, thereby enabling the dose adjustment of TAC based on the dose of MMF. The estimated value of interaction parameter was 0.06 in inverse exponential equation. The CL*/F* of TAC is affected by the interaction parameter as well as the IIV of CL*/F* and *CYP3A5* genotypes. To evaluate the clinical significance of the interaction in a collective manner, we simulated the model under the various clinical scenarios. As presented in Table 3, the required dose of TAC changed by 0.5 mg when the dose of MMF changes by 250–500 mg. Moreover, the effect of interaction between TAC and MMF was in line with previous *in vitro* and clinical studies. A previous *in vitro* study also observed the inhibition of TAC metabolism when incubated with MPA^14^. In liver transplant recipients, AUC of TAC was increased by approximately 20% when MMF was co-administered^35^.

On the other hand, research by Kagaya, *et al*. insisted that there is no DDI between TAC and MPA in renal transplant recipients^36^. In that study, patients were divided into five groups according to AUC of MPA and pharmacokinetic parameters of TAC were compared between groups. Statistical comparison according to genotypes (*CYP3A5* and *UGT2B7*) were also done. However, the analyses results according to the AUC of MPA or genotypes were done separately, therefore the results were not directly comparable to those in our study. Another recent research by Rong, *et al*. showed the lack of interaction between TAC and MMF based on their multiple analysis results including the population covariate modeling, multiple regression, and categorical analysis^37^. However, the most notable difference of our approach is the use of the integrated model-based approach in our study. The integrated modeling approach enables the simultaneous consideration of clinical covariates including genotypes and concentrations of co-administered drugs. The combined effect of co-administered MMF and *CYP3A5* genotype was captured in our study.

In clinical practice, the administration of MMF frequently changes due to an adverse event or infection episodes affecting the dose requirement of TAC. Because of TAC’s narrow therapeutic index and high IIV in dose-response especially in patients taking MMF concurrently, the current dosing approach may not be an adequate strategy as it neglects the impacts of genetic variability on the system’s ability and drug interaction of MMF. For this reason, the pharmacokinetic model for dosing algorithm are useful to aid the flexible adjustment of TAC exposure in response to the change in the administration of MMF according to *CYP3A5* genotype. Although some studies developed dosing algorithms of TAC, those algorithms are limited because of fact that they have focused on determining the starting dose or ignored the effect of interaction with co-administered MMF^20,38^. Our study results suggest prescribers to consider monitoring the level of TAC when they initiate or increase the dose of MMF especially in *CYP3A5* non-expressers. As a higher level of TAC is a risk for nephrotoxicity, neurotoxicity, or infection and a lower level of TAC is directly linked to acute rejection, this model can aid meticulous maintenance of TAC exposure in both *CYP3A5* expressers and non-expressers^39,40^.

The current research has few limitations. Because the model was developed using patients with a median of 5.7 years post-transplant, their HCT or GFR levels were near normal. Due to their low variability, the model did not capture the known effects of HCT and GFR on the pharmacokinetics of TAC and MPA. Therefore, the predictive performance of the developed model might be limited to patients who maintain a stable HCT and graft function. Secondly, though the exposure-response relationship of MMF is known to be nonlinear^41^, we failed to characterize a nonlinear aspect into the model equation. Small dose range width of administered MMF in the study population might explain the reason. Future work is required to generalize the results to more variable situations including the unstable early period after transplantation or the higher dose of TAC or MMF.

In summary, the interaction effect between TAC and MMF was evaluated in kidney transplant recipients. In the final population pharmacokinetic model, *CYP3A5* genotype and co-administration with MMF were identified as significant factors in determining the CL*/F* of TAC. The effect of co-administered MMF in determining the level of TAC exposure is more pronounced in *CYP3A5* non-expressers. The structure model explaining the interaction between TAC and MMF can also be served as the reference structure in other clinical situations including an early period after transplantation. By considering the *CYP3A5*-mediated DDI between TAC and MMF, personalized dose adjustment in accordance with dosing algorithm can be applied in a maintenance period after transplantation. Improvement in post-transplant management including rejection prevention is expected through better maintenance of the TAC concentration in the target range especially in patients taking interacting drugs.

## Methods

### Study design

The study was designed to assess the pharmacokinetic interaction by serial sampling. Eligible patients took their medications as usual. Inclusion criteria of the study were as follows: (1) at least six months after kidney transplant; (2) oral administration of study drugs, TAC (Prograf^®^) and MMF (Cellcept^®^); and (3) maintained the same dosage and administration methods for at least two weeks prior to the study (for both TAC and MMF). Exclusion criteria were as follows: (1) multi-organ transplantations; (2) gastrointestinal disorders that may affect the absorption of the study drugs; (3) had taken other drugs that may strongly affect the pharmacokinetics of the study drugs; and (4) abnormal liver function with AST or ALT > 3x the upper limit of normal range.

The study was conducted in compliance with the Declaration of Helsinki, the International Conference on Harmonization Guidelines for Good Clinical Practice^42^. This study was approved by the institutional review board (IRB No. C-1604-014-753) of Seoul National University Hospital (Seoul, Korea). All subjects were given written informed consent (Clinicaltrials.gov identifier NCT02808065).

### Bioanalytical methods

Blood samples were drawn pre-dose and post-dose 1, 2, 3, and 4 hours after taking TAC and MMF at the same time. Sample storage and analytical methods were described previously^23^. Briefly, concentrations of TAC were measured in whole blood with the validated liquid chromatography/tandem mass spectrometry (LC-MS/MS). Plasma concentrations of MPA, MPA 7-O-glucuronide (MPAG), and MPA acyl glucuronide (AcMPAG) were simultaneously analyzed with LC-MS/MS. Plasma was acidified with phosphoric acid (850 g/L; Sigma-Aldrich) at the time of blood specimen collection^23^. Concentrations of analytes were linear and accurate in the range of analysis with the coefficient of variation (CV) less than 6%. Lower limit of quantification for TAC, MPA, MPAG, and AcMPAG was 0.5 ng/mL, 0.1 µg/mL, 0.1 µg/mL, and 0.2 µg/mL, respectively. Genotypes including CYP3A4*1G (rs2242480), CYP3A5*3 (rs776746), SLCO1B1*1B (rs2306283), SLCO1B1*5 (rs4149056), *SLCO1B3* 334T > G (rs4149117), *SLCO1B3* 699G > A (rs7311358), *ABCC2* –24C > T (rs717620), *ABCC2* 1249G > A (rs2273697), *ABCC2* 3972C > T (rs3740066), UGT1A9*1b (rs3832043), and *UGT2B7* 802C > T (rs7439366) were determined. Methods for genotyping were also described in our previous research^23^.

### Clinical data collection

Demographic variables including age, sex, weight, height, post-operative days, the levels of hemoglobin, HCT, serum creatinine, estimated glomerular filtration rate (eGFR) calculated by Modification of Diet in Renal Disease (MDRD) equation^43^, albumin, total bilirubin, and electrolytes were collected at the time of the blood sampling. Recent administration history of study drugs and concomitant medications were also collected.

### Population pharmacokinetic model development

**Figure 2:**
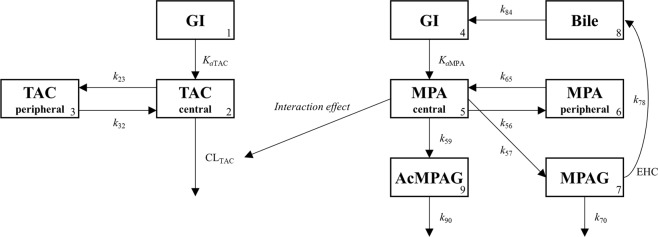
Schematic presentation of a population pharmacokinetic model. Compartments: gastrointestinal tract (GI, 1, 4), central compartment for tacrolimus (2), peripheral compartment for tacrolimus (3), central compartment for mycophenolic acid (5), peripheral compartment for mycophenolic acid (6), compartment for mycophenolic acid 7-O-glucuronide (7), compartment for gall bladder (8), compartment for mycophenolic acid acyl glucuronide (9). TAC, tacrolimus; *K*_a_, absorption rate constant; *k*_23_, *k*_32_, *k*_56_, and *k*_65_, intercompartment rate constants; *CL*, clearance; MPA, mycophenolic acid; MPAG, MPA 7-O-glucuronide; AcMPAG, MPA acyl glucuronide; *k*_57_ and *k*_59_, metabolized rate constants for mycophenolic acid; EHC, enterohepatic circulation; *k*_78_, biliary recirculation of MPAG into GI; *k*_70_ and *k*_90_, eliminated rate constants; *k*_84_, gallbladder emptying rate constant; Meal times were used to trigger timing of gall bladder emptying.

Population pharmacokinetic model was developed to estimate pharmacokinetic parameters of TAC and MMF in consideration of the effect of interaction and clinical covariates. Population pharmacokinetic parameter estimates were obtained by using user defined subroutine ADVAN6. Model development procedure consisted of sequential steps of structure model building, explanation of the residual error, and identification of significant covariates. The structure model from previous research, which has characterized the interaction effect between TAC and MMF with an inverse exponential equation, was fitted to observed concentrations from kidney transplant recipients^23^. This is presented in Fig. 2. The pharmacokinetics of TAC was explained by two-compartment, first-order absorption with lag time, and first-order elimination. The structure model of MMF included compartments of MPA, MPAG, AcMPAG, and gallbladder. Pharmacokinetics of MPA was described by two-compartment and first-order absorption. Elimination route of MPA was limited to the metabolic clearance to MPAG and AcMPAG. Enterohepatic circulation was modeled with the gallbladder compartment and a mass transfer rate constant between the gallbladder and the gastrointestinal tract. Model event time parameter (MTIME) was introduced to control the transfer of bile acid from the gallbladder into the gastrointestinal tract. Because of the relatively short period of blood sampling window, it was not enough to fully characterize the absorption and enterohepatic circulation process of study drugs. Therefore, pharmacokinetic parameters related to absorption (absorption rate constant and lag time) and enterohepatic circulation (percentage of enterohepatic circulation, transfer rate constant, and model event time) were fixed to values from the population pharmacokinetic models for healthy volunteers^23^. Effect of interaction was modelled as inverse proportional model as was in healthy volunteer^23^.

IIV in pharmacokinetic parameters was assumed for apparent clearance and volume of distribution of TAC and MPA. Residual error was assessed as an additive, proportional, or combined additive and proportional model.

After the development of the structure model, the significance of covariates was tested. Continuous covariates including body weight, age, serum creatinine, MDRD eGFR, and HCT were centered on the median and categorical covariates like genotypes were tested as a binary variable. The effect of covariates was explored with a stepwise covariate modeling procedure. On the other hand, the statistical significance of model with covariates was determined based on the difference between OFVs which were computed by the log-likelihood ratio test. In case of forward inclusion, the effect of included covariate was regarded significant if an objective function value decreased by more than 3.84 (*p* < 0.05). In backward elimination procedure, if covariates were included and the OFVs increased to more than 6.63 (*p* < 0.01), the corresponding covariates were excluded.

NONMEM version 7.3 (ICON Development Solutions, Hanover, MD) was used to develop a population pharmacokinetic model. Perl-speaks-NONMEM version 4.4.8, Xpose 4, and R version 3.2.2 were used to aid a modeling process and to generate graphical outputs^44,45^.

### Model evaluation

In the process of population pharmacokinetic model development, various model evaluation methods were applied. Scientific plausibility of final estimates, RSE of estimates, objective function value, and shrinkage were evaluated to test the appropriateness of the model^46,47^. The goodness-of-fit plot was used to assess model fit and distribution of residuals^46^. Predictive performance of the model was evaluated with a prediction-corrected VPC^48^.

### Model simulation

Thirty-two sets of the virtual population (*n* = 1,000) were generated according to the combination of TAC dose (1 mg bid, 2 mg bid), MMF dose (500 mg bid, 1,000 mg bid), and possible states of identified clinical covariates. In each population, concentrations of TAC were simulated with the final population pharmacokinetic model. C/D of TAC at the steady state were compared among the virtual populations. Linear regression analysis was done to estimate the effect of covariates on C/D of TAC. Estimated effect was presented as percent increase from the baseline level of C/D of TAC (intercept). With considering the effect of significant covariates, required dose of TAC was simulated under the different clinical circumstances and the dosing algorithm of TAC was developed upon. The recommended daily dose of TAC was determined to include more simulated concentrations in the target range.

## Supplementary information

Supplementary Information
