# Supplementary material for: Model based development of tacrolimus dosing algorithm considering CYP3A5 genotypes and mycophenolate mofetil drug interaction in stable kidney transplant recipients
Source: Sci Rep. 2019 Aug 13;9:11740. doi: 10.1038/s41598-019-47876-0 (PMC6692323; doi:10.1038/s41598-019-47876-0)
Supplement: Supplementary file 1 — Supplementary Information [file 41598_2019_47876_MOESM1_ESM.pdf]

Model based development of tacrolimus dosing algorithm considering CYP3A5 genotypes and mycophenolate mofetil drug interaction in stable kidney transplant recipients

Jae Hyun Kim Ph.D.[1], Nayoung Han Ph.D.[1], Myeong Gyu Kim Ph.D.[1,2], Young Won Kim M.S.[1], Hayoung Jang B.S.[1], Hwi-Yeol Yun Ph.D.[3], Mi-Yeon Yu M.D.[4], In-Wha Kim Ph.D.[1], Yon Su Kim M.D., Ph.D.[5,6*] and Jung Mi Oh Pharm.D.[1*]

[1]College of Pharmacy and Research Institute of Pharmaceutical Sciences, Seoul National University, Seoul, Republic of Korea

[2]Graduate School of Clinical Pharmacy, CHA University, Republic of Korea

[3]College of Pharmacy, Chungnam National University, Daejeon, Republic of Korea

[4]Department of Internal Medicine, Hanyang University Guri Hospital, Guri, Republic of Korea

[5]Kidney Research Institute, Seoul National University College of Medicine, Seoul, Republic of Korea

[6]Department of Medical Science, Seoul National University College of Medicine, Seoul, Republic of Korea

***Co-corresponding Authors**

Yon Su Kim M.D., Ph.D.

Department of Medical Science, Seoul National University College of Medicine, 101 Daehak-ro, Jongno-gu, Seoul 03080, Korea.

Kidney Research Institute, Seoul National University College of Medicine

101 Daehak-ro, Jongno-gu, Seoul 03080, Korea

Tel.: +82 2 2072 2264; fax: +82 2 745 2264; email address: yonsukim@snu.ac.kr

Jung Mi Oh Pharm.D., College of Pharmacy, Research Institute of Pharmaceutical Sciences, Seoul National University, 1 Gwanakro, Gwanak-gu, Seoul 08826, Korea. Tel.: +82 2 880 7736; fax: +82 2 882 9560; email address: jmoh@snu.ac.kr

Figure legend

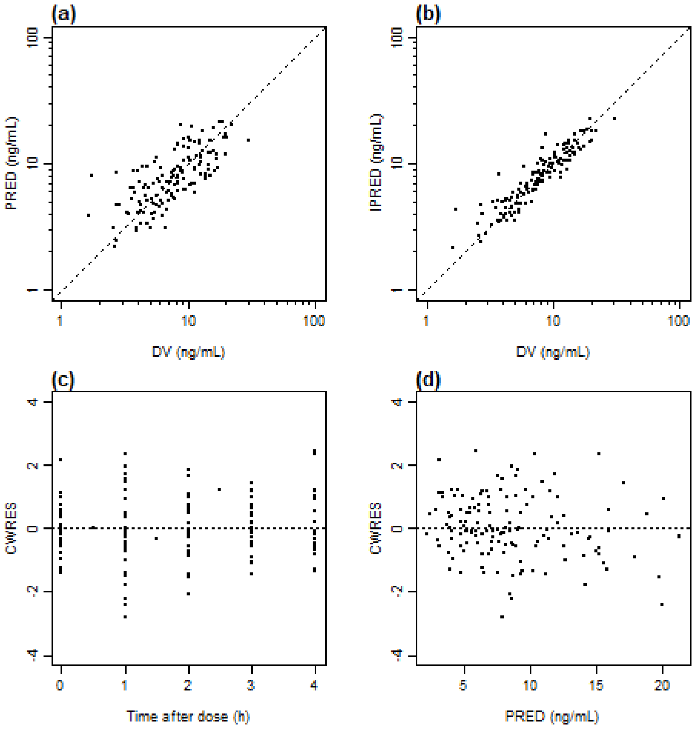

Supplementary Figure S1 Goodness-of-fit plot of tacrolimus for the population pharmacokinetic model. (a) population prediction (PRED) *vs.* observed concentration (DV); (b) individual prediction (IPRED) *vs.* DV; (c) conditional weighted residuals (CWRES) *vs.* TIME; (d) CWRES *vs.* PRED.

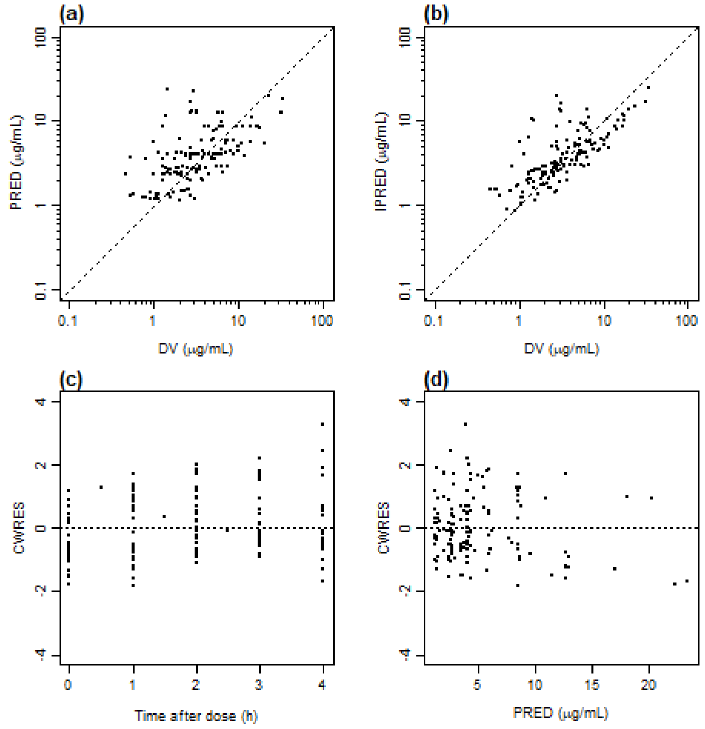

Supplementary Figure S2 Goodness-of-fit plot of mycophenolic acid for the population pharmacokinetic model. (a) population prediction (PRED) *vs.* observed concentration (DV); (b) individual prediction (IPRED) *vs.* DV; (c) conditional weighted residuals (CWRES) *vs.* TIME; (d) CWRES *vs.* PRED.

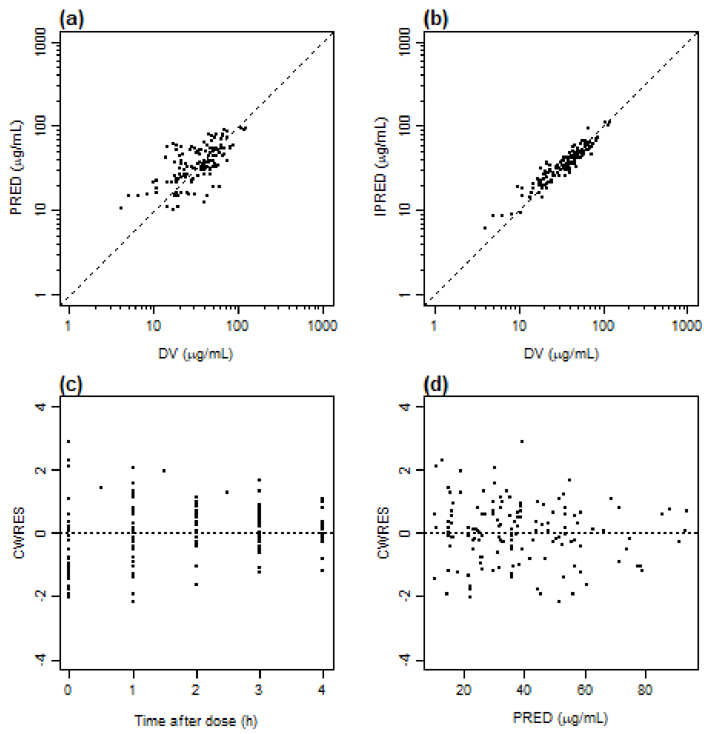

Supplementary Figure S3 Goodness-of-fit plot of mycophenolic acid 7-O-glucuronide for the population pharmacokinetic model. (a) population prediction (PRED) *vs.* observed concentration (DV); (b) individual prediction (IPRED) *vs.* DV; (c) conditional weighted residuals (CWRES) *vs.* TIME; (d) CWRES *vs.* PRED.

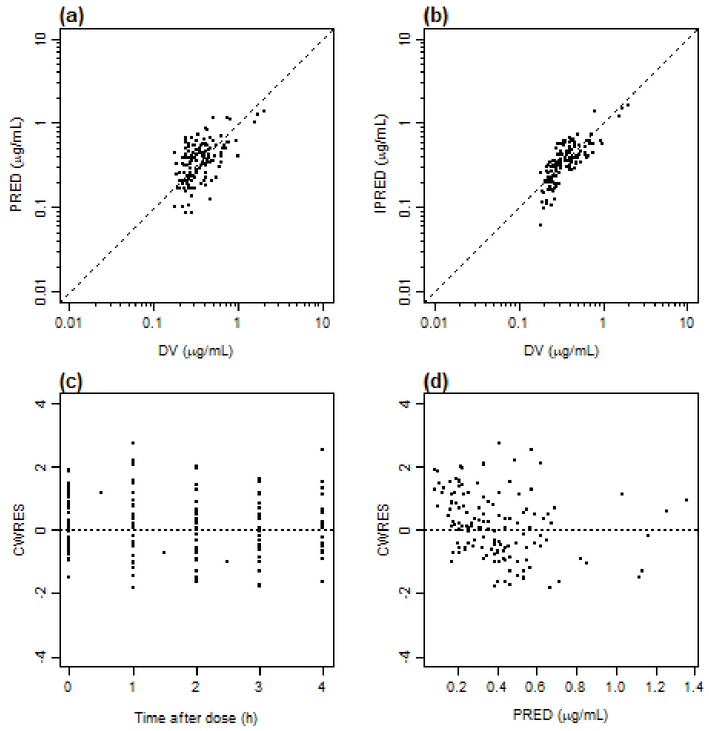

Supplementary Figure S4 Goodness-of-fit plot of mycophenolic acid acyl glucuronide for the population pharmacokinetic model. (a) population prediction (PRED) *vs.* observed concentration (DV); (b) individual prediction (IPRED) *vs.* DV; (c) conditional weighted residuals (CWRES) *vs.* TIME; (d) CWRES *vs.* PRED.
